# Laboratory-confirmed sexually transmitted infections and associated factors among street-based female sex workers in Southwest Ethiopia: a facility-based cross-sectional study

**DOI:** 10.1186/s12889-026-28539-y

**Published:** 2026-07-14

**Authors:** Dawit Haile Zeben, Getachew Yitayew Tarekegn, Bekana Fekecha Hurissa, Taju Mohamed A/Macha, Hassen A/Raya A/Jobir, Mudin Hussen A/Bor, Genzebe Asfaw Lakew, Bosena Tebeje Gashaw

**Affiliations:** 1 Department of Midwifery Education, School of Midwifery, Institute of Health, Jimma University, P.O. Box 378, Jimma, Ethiopia; 2 Department of Pharmacy, College of Health Sciences, Debre Tabor University, Debre Tabor, Ethiopia; 3 Department of Maternity and Reproductive Health, School of Midwifery, Institute of Health, Jimma University, Jimma, Ethiopia; 4 Department of Nursing, Institute of Health, Jimma University Medical Center, Jimma University, Jimma, Ethiopia; 5 Department of Business Management, School of Business, Rift Valley University, Jimma, Ethiopia; 6 Department of Mechanical Engineering, Jimma University, Jimma, Ethiopia; 7 Department of Pharmacy, College of Pharmacy, Rift Valley University, Jimma Branch, Jimma, Ethiopia

**Keywords:** Sexually transmitted infections, Street-based sex work, Female sex workers, Jimma, Ethiopia

## Abstract

**Background:**

Street-based sex work raises women’s risks regarding sexual health, particularly concerning STIs, due to factors such as violence, limited condom negotiation power, and inadequate access to health services. Evidence on STIs among female sex workers in Southwest Ethiopia is notably scarce, despite the public health implications.

**Objective:**

To assess the prevalence of laboratory-confirmed sexually transmitted infections and associated factors among street-based female sex workers in Southwest Ethiopia.

**Methods:**

A facility-based cross-sectional study was conducted among 203 street-based female sex workers attending key population clinics in Jimma and Agaro key population clinic towns. Data were collected using structured face-to-face interviews, and biological samples were analyzed for HIV, gonorrhea, chlamydia, and syphilis using standard serological and nucleic acid amplification tests in accordance with national STI testing algorithms at three key population clinics from March to December 2025. To avoid duplicate enrollment during the prolonged study period, unique identification codes and clinic records were used to ensure that each participant was included only once. Data were analyzed using SPSS version 26, and variables with *P* ≤ 0.25 in the bivariable analysis were entered into the multivariable logistic regression model.

**Results:**

Overall, 38.9% (95% CI: 32.2–45.8) of participants had at least one laboratory-confirmed STI. The prevalence of HIV, syphilis, gonorrhea, and chlamydia was 10.3%, 11.0%, 8.4%, and 8.9%, respectively. More than half (54%) reported initiating sex work before age 18. Early initiation of sex work was independently associated with laboratory-confirmed STIs (AOR = 3.16, 95% CI, 2.02–7.22). Other significant predictors included illiteracy (AOR = 2.34, 95% CI: 1.22–6.35), rural origin (AOR = 2.90, 95% CI: 1.57–5.81), having more than two clients per day (AOR = 2.55, 95% CI: 1.32–4.95), inconsistent condom use (AOR = 3.03, 95% CI: 2.14–5.93), and experience of sexual violence (AOR = 2.77, 95% CI: 1.48–5.20).

**Conclusion:**

The prevalence of laboratory-confirmed STIs was high among street-based female sex workers in southwest Ethiopia. Early initiation of sex work, low educational status, rural origin, high client numbers, inconsistent condom use, sexual violence, and limited access to services were independently associated with increased STI risk. These findings highlight the need for integrated, targeted interventions focusing on prevention, improved service access, and structural vulnerabilities.

**Supplementary Information:**

The online version contains supplementary material available at https://doi.org/10.1186/s12889-026-28539-y.

## Background

Sexually transmitted infections (STIs) remain a major public health problem worldwide, with a great burden that is not evenly distributed across populations. Globally, over one million curable STIs are acquired every day, and key populations, such as female sex workers (FSWs), are disproportionately affected. Across settings worldwide, women at higher risk have higher rates of HIV, syphilis, gonorrhea, and chlamydia than women in the general population, partly due to increased exposure to infection, patterns of sexual behavior, and structural barriers to prevention and care. In sub-Saharan Africa, studies consistently show that HIV prevalence among FSWs is about 10–30 times higher than that among the general female population, reflecting the concentrated epidemic in the region [1–3].

Early entry into sex work, usually defined as starting before 18 years of age, is linked to poorer sexual and reproductive health outcomes [4]. Adolescents who enter sex work at a younger age face higher biological vulnerability to infection and often have limited knowledge, limited power to negotiate safer sex, and limited access to prevention and health care services [5, 6]. Within diverse environments, investigations reveal that early initiation into sex work is linked to inconsistent condom use, a higher number of clients, substance use, and more frequent physical and sexual violence, which together increase the likelihood of acquiring STIs [7, 8]. Street-based sex work is widely considered one of the most marginalized and high-risk forms of sex work, and these can be especially acute for people who are also parents [9]. Women engaged in street base sex work often experience unsafe working environments, stigma, violence, unstable income, and limited access to health services [10]. Risk tends to be higher for women who enter sex work at a young age, especially in low- and middle-income countries where social protection systems are limited [11].

In Ethiopia, sex work occurs in both establishment-based and street-based sex settings, and street-based work is more common in urban and per-urban areas [12]. Evidence from Ethiopia is consistent with that many female sex workers enter sex work during adolescence, often driven by poverty, limited education, migration, family disruption, and other socioeconomic vulnerabilities [13–15]. Although STIs have received increasing attention in Ethiopia, national guidelines mainly use a syndromic approach for diagnosis and treatment, which may miss asymptomatic infections [16]. Therefore, this laboratory-confirmed diagnosis provides a more accurate measure of STI infection status and allows a clearer assessment of risk factors, such as age at entry into sex work without laboratory confirmation, understanding of the prevalence of STIs remains poor. This study addresses the gap in knowledge regarding the factors related to STIs among women who begin street-based sex work at an early age.

## Methods

### Study setting, design, and period

This study was conducted in Jimma and Agaro town, Jimma Town, in the Jimma zone of Oromia regional state, which is located 352 km from Addis Ababa and has an estimated population of 500,000 (CSA,2024). The municipality comprises 3 sub-cities, 13 urban kebeles, and 4 rural kebeles, and is served by 128 health facilities, including one specialized hospital, one general hospital, seven private hospitals, four public health centers, 55 private clinics, and multiple pharmacies and drug stores. Agaro, an independent wereda in the same zone, is situated at 1.560 m above sea level and had a population of 52 802 (2022 estimate) from the city population. The cross-sectional study was conducted among street-based female sex workers attending key population clinics in Jimma and Agaro towns from March 20 to December 20, 2025.

### Study population

The study participants were street-based female sex workers who attended key population clinics in Jimma and Agaro towns during the data collection period.

### Inclusion criteria

Women aged ≥ 18, provided written informed consent and agreed to provide biologic undefined samples for STI testing were included in the study.

### Exclusion criteria

Participants were excluded if they were critically ill or unable to respond at the time of data collection, had received antibiotic treatment for a sexually transmitted infection (STI) within the previous weeks. Declined to provide biologic undefined samples, or did not provide informed consent.

### Data collection procedures

**Fig. 1:**
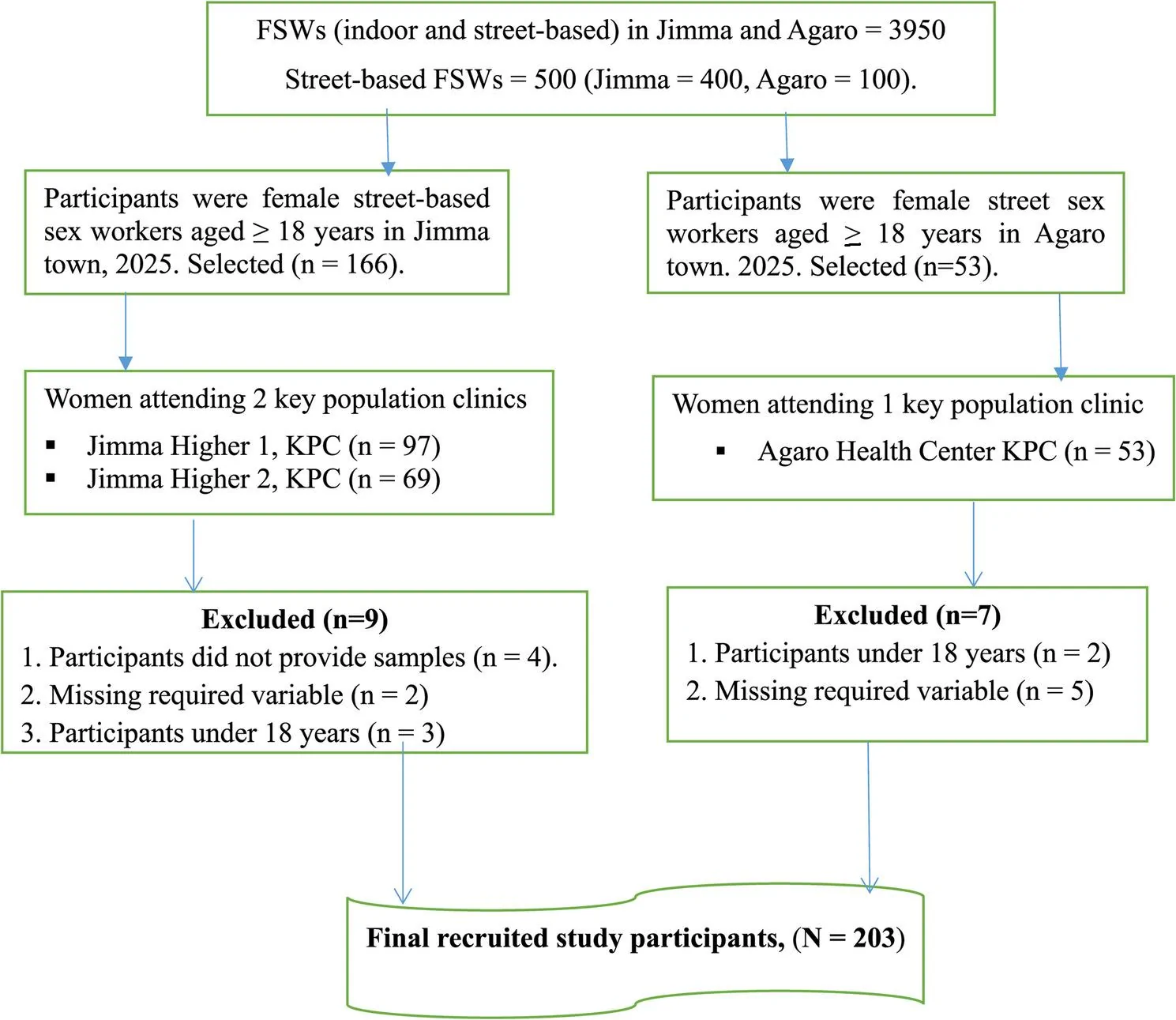
Participant screening, eligibility, and recruitment flowchart for laboratory-confirmed female street-based sex workers in Jimma and Agaro towns. Consecutive sampling was used; all eligible street-based female sex workers attending the key population clinics during the data collection period were included

This study was conducted among street-based female sex workers in Jimma and Agaro towns. From an estimated population of 3,950 female sex workers (3,450 in Jimma and 500 in Agaro), participants were recruited from women attending three key population clinics (Jimma Higher 1 Health Center, Jimma Higher 2 Health Center, and Agaro Health Center). Using consecutive sampling, all women who met the inclusion criteria during the data collection period were screened. Of these, 219 were found eligible, and 203 women were finally recruited from both towns. This study was conducted among street-based female sex workers in Jimma and Agaro towns Fig. 1.

These clinics provide routine sexual and reproductive health services for all FSWs, including street-based sex workers, STI screening, HIV testing, counseling, and condom distribution. Only the SBSWs who visited the clinics during the data collection period were the source population for this study. Participants were recruited using a consecutive sampling approach. Eligible women were identified and classified as street-based sex workers by trained health care centers at the key population clinics, based on national service delivery guidelines [12, 17]. Accessed the clinics through peer referrals or self-presentation for routine services, typically every 3–6 months. Recruitment was conducted proportionally across the three key population clinics, and all eligible participants who provided informed consent were consecutively enrolled throughout the data collection period. Data were collected within the settings using a structured, interviewer-administered questionnaire conducted in private rooms to ensure confidentiality by three trained female BSc nurses. Following the interview, blood and vaginal swab samples were collected by three trained BSc female laboratory professionals for laboratory confirmation of sexually transmitted infections and HIV testing as part of routine clinical care. All procedures followed standard operating procedures, and participants received appropriate counseling, treatment, and referrals in line with national protocols.

### Laboratory testing for STI diagnosis

Laboratory services were provided through the facility’s routine laboratory system. Chlamydia trachomatis was detected using nucleic acid amplification tests (NAATs), while Neisseria gonorrhoeae was detected using Gram staining and standard light microscopy among symptomatic participants through identification of intracellular Gram-negative diplococci in clinical specimens. Gram staining and microscopy were not used for the detection of Chlamydia trachomatis. Syphilis was screened using the rapid plasma reagin (RPR) test and confirmed using treponemal tests. HIV infection was diagnosed according to the Ethiopian national serial rapid diagnostic testing algorithm. The outcome variable was recorded as a binary measure (positive/negative for laboratory-confirmed STI) according to national guidelines and standard operating procedures based on WHO-recommended STI diagnostic approaches. All laboratory procedures were performed by trained laboratory professionals following standard operating procedures. Diagnostic tests were conducted using validated methods and appropriate diagnostic kits. Quality assurance measures included duplicate testing of 5% of samples, the use of positive and negative controls, and independent verification of results by senior laboratory personnel. Laboratory results were linked to participant data using unique identification codes to ensure accurate data integration, minimize measurement bias, and support reliable statistical analysis.

### Study variables

#### Outcome variable

The primary outcome variable was laboratory confirmed sexually transmitted infection (STI), defined as the presence of at least one STI diagnosed through laboratory testing at the time of clinic visit. The outcome was coded as a binary variable (Yes/No).

#### Independent variables

Independent variables were grouped into sociodemographic, behavioral, violence and coercion-related, and reproductive health service-related factors. Sociodemographic variables included age, participants’ educational level, maximum parental educational level, early initiation of sex work, place of origin (rural to urban), duration of sex work, and migration status. Behavioral factors included alcohol use before sexual intercourse, use of khat, shisha, or other psychoactive substances, and frequency of substance use. Violence and coercion – related included self-reported experience of physical violence, experience of sexual violence, and the ability to negotiate condom use with clients. Reproductive health service-related variables included history of STI symptoms in the past 12 months, previous STI diagnoses by health professionals, history of HIV testing, frequency of STI screening, and access to targeted STI prevention and treatment services. These variables were selected based on the existing literature demonstrating their relevance to STI risk among female sex workers.

#### Operational definition and terms

- ▪ Laboratory – confirmed STI: presence of one or more sexually transmitted infections confirmed by laboratory testing at key population clinics during the study period (Yes/No).
- ▪ Street-based sex workers: A women who solicit clients in public places and are identified as such by health care centers at key population clinics.
- ▪ Access to targeted STI prevention and treatment services: was defined as self-reported use of STI–related prevention or treatment services.
- ▪ Early initiation of sex work: Self-reported initiation of sex work before 18 years of age.
- ▪ Key population clinics: A health facility providing targeted STI, HIV testing, counseling, and prevention services for key populations.
- ▪ Alcohol use before sex: Self-reported consumption of alcohol before sexual intercourse with clients in the past 3 months.
- ▪ Substance use: Self-reported use of khat, shisha, or other drugs in the past three months.
- ▪ Physical violence: Self-reported experience of physical assault related to sex work.
- ▪ Sexual violence: Self-reported experience of forced or coerced sexual acts.

#### Data quality assurance, data management, and analysis

To ensure data quality, the questionnaire was adapted from relevant literature [1, 16, 18] and pretested before data collection. Data collectors and supervisors received standardized training on study objectives, interview techniques, confidentiality, and ethical considerations. Interviews were conducted in private rooms to minimize information bias, and completed questionnaires were reviewed daily by supervisors for completeness and consistency. Laboratory procedures followed standard operating guidelines, and quality control measures were applied throughout specimen collection, handling, and testing. Data were entered using Epi-Data version 4.6 and exported to SPSS version 26 for analysis. Data cleaning was performed to check for missing values, inconsistencies, and outliers. All statistical analyses were conducted using SPSS version 26. All electronic data were password protected, and access was limited to the research team to maintain confidentiality. Data analysis involved descriptive statistics to summarize participants’ characteristics and the prevalence of laboratory-confirmed STIs. Bivariate logistic regression was used to assess the association between independent variables and laboratory-confirmed STI status. Variables with a p-value < 0.25 in the bivariable analysis were considered candidates for the multivariable logistic regression model. Accordingly, only variables meeting this predefined criterion were included in the multivariable analysis. Adjusted odds ratios with 95% confidence intervals were reported, and statistical significance was declared at *P* < 0.05. Potential confounders were selected based on prior literature and variables with *P* < 0.25 in bivariable analysis. Multicollinearity was assessed using variance inflation factor (VIF). Model diagnostics included the Hosmer – Lemeshaw goodness-of-fit, checks for sparse cells, and estimates of stability.

#### Missing data handling

The study used a strict method for handling data, which included standardized questionnaires and a comprehensive quality control process. These included methodological completeness checks, interview training, and pretesting of instruments and biologic undefined sample tests. Tools like SPSS were used for statistical analysis, whereas specialist software was used for data entry and cleaning. Missing data were assessed for completeness, and cases with substantial missing variables were excluded from the final analysis. No imputation method was applied.

## Results

### Socio-demographic factors

This study achieved a 92.7% rate with 203 respondents. More than half of the participants had early initiation of street-based sex work, 38.4% had rural place of origin, almost half (47.8%) had less than one year remained in this work, and 35% of participants were illiterate (Table 1).

**Table 1:** Sociodemographic characteristics of female street-based sex workers by laboratory-confirmed STI status in Jimma and Agaro towns, Southwest Ethiopia, 2025 (*n* = 203)

Category of variable	Frequency (*n* = 203)	Percentage (%)
Age of participants ( in years)		
18–24	119	58.6
25–29	66	32.5
≥ 30	18	8.9
Educational status of the participants		
Illiterate	73	35
Primary	100	49.2
Secondary and above	30	14.8
Educational status of parents		
Illiterate	92	45.3
Primary	79	38.9
Secondary and above	32	15.7
Early initiation of SBSW (< 18 years)		
Yes	110	54.2
No	93	45.8
Place of origin		
Rural	78	38.4
Urban	125	61.6
Migration		
Yes	83	40.8
No	120	59.2
Duration of sex work (years)		
< 1years	97	47.8
1–3 years	63	31
≥ 3 years	43	21.2

### Substance use-related factors

Out of the 203 participants, 76 (37.4%) consumed alcohol weekly. Moreover, 81 (40.0%) always reported using alcohol, and 53 (26.1%) sometimes reported using alcohol before sex. In addition, 48.3% reported using khat and shisha, and about 10.8% used hard drugs, including cocaine and tranquilizers (Table 2).

**Table 2:** Substance use risk related to sexual transmission infection of female street-based sex workers in Jimma and Agar Towns, Oromia Region, Southwest, Ethiopia 2025 (*n* = 203)

Category of variable	Frequency (*n* = 203)	Percentage (%)
Alcohol use before sex		
Always	81	40
Sometimes	53	26.1
Never	69	33.9
Use of khat, shisha, or other drugs		
Yes	98	48.3
No	105	51.7
Frequency of alcohol use		
Daily	87	42.9
Weekly	76	37.4
Occasionally	40	19.7

### Sexual risk behavior-related factors

From the total number of participants, 120 (59.1%) reported experiencing condom breakage or slippage in the last three months. In addition, 142 participants (70%) averaged more than two clients per day. However, only 15.2% of participants used condoms consistently with their clients in the last seven days, and only 13.3% did so over the past 12 months (Table 3).

**Table 3:** Sexual risk-related factors of sexual transmission infection of female street-based sex workers in Jimma and Agaro Towns, Oromia Region, Southwest, Ethiopia 2025 (*n* = 203)

Category of variable	Frequency (*n* = 203)	Percentage (%)
Average number of clients per day		
< 2 clients/day	61	30
More than 2 clients/day	142	70
Condom use with clients in the last 7 days		
Never	83	41
Sometimes	89	43.8
Always	31	15.2
Condom breakage or slippage in the last 3 months		
Yes	120	59.1
No	83	40.9
Consistent condom use in the past 12 months		
Yes	27	13.3
No	176	86.7

### Violence and coercion-related factors

Of 82 (40.4) participants reporting experiencing physical violence related to sex work, 66 (32.5%) reported sexual violence or forced sex. Others, most participants 160 (78.8%), reported inability to negotiate condoms with their clients Table 4.

**Table 4:** Prevalence associated with violence and coercion about sexually transmitted infection among female SBSWs in Jimma and Agaro towns, Oromia, southwest Ethiopia, 2025 (*n* = 203)

Category of variable	Frequency (*n* = 203)	Percentage (%)
Experience of physical violence related to sex work.		
Yes	82	40.4
No	121	59.6
Experience of sexual violence or forced labor		
Yes	66	32.5
No	137	67.5
Ability to negotiate condom use with clients.		
Yes	43	21.2
No	160	78.8

### Reproductive and health service use

Only 15.3% had been clinically diagnosed with an STI. Less than half (44.8%) had ever been tested for HIV, and nearly half (49.3%) had never undergone STI screening. Access to targeted STI Services was limited to 25.6% of participants. Additionally, 56.1% reported a history of STI symptoms in the past 12 months, while only 19 (9.4%) participants reported current STI-related symptoms during data collection (Table 5).

**Table 5:** Factors of reproductive and health services for sexually transmitted infections among female street-based sex workers in jimma and agaro towns, Oromia region, southwest Ethiopia, 2025 (*n* = 203)

Category of variable	Frequency (*n* = 203)	Percentage (%)
History of STI symptoms in the past 12 months		
Yes	114	56.1
No	89	43.9
Previous STI diagnosis by a health professional		
Yes	31	15.3
No	172	84.7
Ever been tested for HIV?		
Yes	91	44.8
No	112	55.2
Frequency of STI screening		
Every 3 months	14	6.9
Every 6 months	24	11.8
Once a year	65	32
Never	100	49.3
Access to targeted STI services for sex workers		
Yes	52	25.6
No	151	74.4
Current STI-related symptoms during data collection		
Yes	19	9.4
No	184	90.6

During data collection, 19(9.4%) participants reported at least one current STI-related symptom. Abnormal vaginal discharge was the most commonly reported symptom, followed by lower abdominal pain, genital itching, and dysuria (Table 6).

**Table 6:** Current STI-related symptoms reported by participants during data collection among female street-based sex workers in jimma and Agaro towns, Oromia Region, southwest Ethiopia, 2025 (*n* = 19)

Reported STI-related symptom	Frequency (*n*)	Percentage (%)
Abnormal Vaginal discharge	7	36.8
Genital itching	4	21.1
Dysuria	3	15.8
Lower abdominal pains	5	26.3

Percentages are calculated among participants who reported current STI-related symptoms during data collection (*n* = 19). Participants could report more than one symptom

### Laboratory results

Laboratory testing showed that 79 (38.9%) participants had at least one sexually transmitted infection (STI), while 124 (61.1%) tested negative. The prevalence of HIV was 21 (10.3%). Syphilis was detected in 23 (11.3%) participants, while gonorrhea and chlamydia were identified in 17 (8.4%) and 18 (8.9%) participants, respectively. Among participants with laboratory-confirmed STI, 62 (78.5%) had a single infection, whereas 17 (21.5%) had multiple concurrent infections (Table 7).

**Table 7:** Results of laboratory-based sexually transmitted infections among early street-based sex workers in Jimma and Agaro towns, Oromia region, southwest Ethiopia, (*n* = 203), 2025

Category of variable	Frequency (*n* = 203)	Percentage (%)
STI result		
Positive	79	38.9
Negative	124	61.1
HIV test results		
Positive	21	10.3
Negative	182	89.7
Syphilis(RPR)		
Positive	23	11.3
Negative	180	88.7
Gonorrhea test result		
Positive	17	8.4
Negative	186	91.6
Chlamydia test results		
Positive	18	8.9
Negative	185	91.1
Number of STIs per participant		
Single infection	62	78.5
Multiple infection	17	21.5

Prevalence of laboratory-confirmed STIs among female street-based sex workers in Jimma and Agaro towns.

The overall prevalence of laboratory-confirmed sexually transmitted infections was 38.9% (95% CI, 32.2–45.8). The mean age of entry into sex work was 19.8 (± SD 0.95) years; it was 15.6 (± SD 0.49) years among those who initiated before 18 years and 25.4 (± SD 0.9) years among those who initiated at 18 years or older. Variables that met the predefined criteria in the bivariable analysis were entered into the multivariable logistic regression model. Educational status, parenteral education, early initiation of sex work, place of origin, alcohol use before sex, number of clients per day, condom use, and experience of sexual violence (Table 8).

**Table 8:** Bivariate and multivariate logistic regression analyses of factors associated with laboratory-confirmed sexually transmitted infections among female street-based sex workers in Jimma and Agaro towns, southwest Ethiopia (*n* = 203)

Variables	Laboratory confirmed STI status		COR (95%CI)	AOR (95%CI)	*p* value
	STI (+)	STI (-)			
Demographic Variable					
Educational level of the participants					
Illiterate	42 (57.5) 31 (42.5)		2.59 (1.67–7.47)	2.34(1.22–6.35)	0.002
Primary	31 (31.0) 69 (69.0)		2.37(1.78–7.21)	2.05(1.10–6.24)	0.038
Secondary and above	6 (20.0) 24 (80.0)		1.00	1.00	
Maximum educational level of parents					
Illiterate	49 (53.3) 43 (46.7)		2.25(1.38–6.82)	2.92 (1.31–6.49)	0.017
Primary	31 (39.2) 48 (60.8)		1.23 (1.15–5.93)	2.50 (1.11–5.70)	0.040
Secondary and above	11 (34.4) 21 (65.6 )		1.00	1.00	
Early initiation of SBSW (< 18 years)					
Yes	51 (46.4) 59 (53.6)		2.00(1.25–4.16)	3.16 (2.02–7.22)	< 0.001
No	28 (30.1) 65 (69.9)		1.00	1.00	
Place of Origin					
Rural	47 (60.3) 31 (39.7)		4.40(2.38–8.08)	2.90 (1.57–5.81)	0.025
Urban	32 (25.6) 93 (74.4)		1.00	1.00	
Substance use-related factors					
Alcohol use before sex					
Always	36 (44.4) 45(55.6)		2.10(1.73–7.89)	3.20 (1.60–6.37)	0.011
Sometimes	24 (45.3) 29 (54.7)		2.18(1.49–6.96)	2.91 (1.75–6.30)	0.034
Never	19 (27.5) 50 (72.5)		1.00	1.00	
Sexual risk behavior-related factors					
Number of clients per day					
< 2 clients/day	34 (55.7) 27 (44.3)		1.00	1.00	
More than 2 clients/day	45 (31.7) 97 (68.3)		2.70(1.46–5.00)	2.55 (1.32–4.95)	0.046
Condom use with clients in the last seven days					
Never	59 (71.0) 24 (28.9)		3.89(1.52–9.39)	3.03 (2.14–5.93)	0.009
Sometimes	38 (42.7) 51 (57.3)		1.82(0.95–3.49)	2.48 (1.30–4.75)	0.022
Always	12 (38.7) 19 (61.3)		1.00	1.00	
Violence and coercion-related factors					
Experience of sexual violence or forced sex					
Yes	41 (62.1) 25 (37.9)		4.27(2.27–8.01)	2.77 (1.48–5.20)	< 0.001
No	38 (27.7) 99 (72.3)		1.00	1.00	
Reproductive and health service use					
Access to targeted STI services for sex workers					
No	33 (63.5) 19 (36.5)		3.96(1.99–7.83)	3.04 (2.02–7.73)	0.018
Yes	46 (30.5) 105 (69.5)		1.00	1.00	
Frequency of STI screening					
Never	39 (39.0) 61 (61.0)		2.35 (0.62–8.92)	2.40 (1.12–5.52)	< 0.001
Once a year	31 (47.0) 36 (63.0)		3.23 (0.84–12.86)	2.67 (1.38–7.88)	0.003
Every 6 months	5 (20.8) 19 (79.2)		0.96(0.19–4.84)	0.96 (0.19–4.84)	0.572
Every 3 months	3 (21.4) 11 (78.6)		1.00	1.00	

Illiterate women were 2.34 times more likely to have a sexually transmitted infection than younger literate women (AOR = 2.34, 95% CI, 1.22–6.35). Those without formal education and those with less education were 2.92 and 2.50 times more likely to have sexually transmitted infections than individuals with more educated parents (AOR = 2.92, 95% CI: 1.31–6.49) and (AOR = 2.50, 95% CI: 1.11–5.70), respectively. Those who had early initiation to sex work were 3.16 times more likely to have at least one laboratory-based STI/s confirmed than those who did not have early initiation to street-based sex work (AOR = 3.16, 95% CI: 2.02–7.22). Rural-origin women were 2.90 times more likely to be exposed to STI than those from urban origins before entering this study (AOR = 2.90, 95% CI, 1.57–5.81) (Table 8).

## Discussion

In this study, the prevalence of laboratory-confirmed sexually transmitted infections among street-based female sex workers was 38.9% (95% CI, 32.2–45.8). Although only 19 (9.4%) participants reported current STI-related symptoms during data collection, laboratory testing identified STI cases among the study population, suggesting the presence of asymptomatic infections. Therefore, differences in STI prevalence across studies should be interpreted considering symptomatic versus asymptomatic populations and diagnostic methods used. This indicates a considerable burden of infection in this already vulnerable population living in urban and peri-urban settings. This finding is consistent with laboratory-based studies conducted among female sex workers in different settings, where STI prevalence ranged from 13.3 to 43.2% based on biologic testing methods [19].

Furthermore, recent studies using molecular diagnostic techniques for pathogens such as Chlamydia trachomatis and Neisseria gonorrhea have also reported a heavy burden of laboratory-confirmed STIs among female sex workers [18]. The similar prevalence observed in both studies could be further clarified by the use of laboratory-based diagnostic methods, which are more effective in identifying both symptomatic and asymptomatic infections. In addition, both studies focused on female sex workers, a population that shares similar behavioral and structural risk factors. Moreover, the studies were conducted in comparable urban settings where access to consistent STI prevention and treatment services remains limited, contributing to the persistently high burden [1, 20, 21].

Early initiation into sex work (< 18 years) was significantly associated with laboratory confirmed sexually transmitted infections (STIs). This finding is consistent with previous studies that reported a higher likelihood of STIs among female sex workers who entered sex work at a younger age [22]. The participants who started sex work before the age of 18 had about three-fold higher odds of having an STI, as younger entrants often have less power to negotiate condom use and are more likely to be exposed to coercive or unsafe sexual situations. This finding is consistent with studies conducted in sub-Saharan African settings, which reported higher STI risk among individuals who entered the network at an early age [23, 24]. The other associated factors with STIs are the educational background of participants and their parents. Participants with no formal education or only primary education had higher odds of infection than those with secondary education or above. Interestingly, parenteral education showed a similar pattern. These findings agree with studies from Ethiopia and other low- and middle-income countries [10, 14].

Place of origin emerged as another significant determinant of STI risk. The rural-origin street-based sex workers are more than three times more likely to be exposed to the risk of one or more STI infections than urban street-based sex workers. These findings are supported by a prior study that observed that structural vulnerabilities, including migration (rural to urban), poverty, and social marginalization, contribute substantially to STI risk among female sex workers [10]. Behavioral factors, particularly condom use and client volume, were significantly predictors of STI acquisition. Consistent condom use in the past seven days was low, with only 15.2% of participants reporting consistent use. Street-based sex workers who never used condoms with clients had more than twofold higher odds of STI infection than those who consistently used condoms. Those who used condoms sometimes did not show a clear difference in STI risk compared with nonusers. These findings are consistent with global evidence showing that inconsistent condom use increases STI risk and may reflect the cumulative effect of repeated sexual exposure [1, 8].

Similarly, exposure to sexual violence or forced sex was associated with higher STI prevalence, with participants being 2.77 times more likely to have STI infections than nonviolence participants. Alcohol and psychoactive substances can impair judgment, reduce negotiation ability, and increase engagement in high-risk sexual behaviors [25]. Reproductive and health service use among participants was limited, with only a minority accessing targeted STI services and many having never undergone STI screening. Lower engagement with these services was accompanied by a higher occurrence of laboratory-confirmed STIs, whereas women who reported screening that is more regular tended to have lower infection levels [1, 26]. Limited access to sexual health services often leads to late diagnoses and ongoing STI transmission among street-based female sex workers. The evidence indicates that making screening and prevention easier to get and more routine really matters. The findings from Jimma and Agaro show that STIs are not just about one thing. Early sex work, little education, growing up in rural areas, and difficulty reaching health services all stack up to raise risk. This pattern matches what other studies in the region and around the world have found. All in all, it’s obvious: tackling STIs here takes a plan that goes after the bigger picture—structural problems, personal choices, and gaps in health services all at once [27–30].

## Conclusion

This study found a high prevalence of sexually transmitted infections among street-based female sex workers in Jimma and Agaro towns. Several factors were linked to STIs, including early entry into sex work, low educational status, rural background, inconsistent condom use, alcohol use before sex, experience of sexual violence, and limited access to sexual and reproductive health services. The findings indicate the need for improved prevention efforts and better access to targeted health services for this population.

### Recommendation

- ▪ Ministry of Health and regional health bureaus: Strengthen targeted STI screening, treatment, and prevention services for street-based sex workers, prioritizing those who initiated sex work before 18 years. Expand youth- and sex worker-friendly services to reduce stigma and improve access.
- ▪ Zonal and town health offices (Jimma and Agaro): Implement out-reach-based mobile clinics for regular STI screening, condom distributions, and health education in sex work hotspots. Integrate violence screening, referral, and post-violence care into routine sexual health services.
- ▪ Non-governmental and community-based organizations: Deliver peer-led behavioral interventions promoting consistent condom use, substance use reduction, and empowerment for young and newly initiated sex workers. Support basic literacy and livelihood programs to address socioeconomic vulnerability.
- ▪ Educational and social affairs sectors: Strengthen girl’s education retention and social protection programs, particularly for rural migrant girls, to prevent early entry into sex work.
- ▪ Researchers and academic institutions: Conduct longitudinal and intervention-based studies to evaluate targeted STI prevention strategies and inform evidence-based policies.

### Strengths and limitations of the study

A major strength of this study is the use of laboratory-based testing to identify sexually transmitted infections, which provides more reliable estimates than syndromic diagnosis alone. The study also addresses an under-researched and vulnerable population, generating evidence that may be useful for similar settings. Furthermore, data were collected using standardized instruments by trained data collectors, helping to ensure data quality. Several limitations should also be considered. Because the study employed a cross-sectional design, causal relationships cannot be established. The use of facility-based consecutive sampling may limit the generalizability of the findings. In addition, some information was obtained through self-report, making the results susceptible to recall and social desirability bias. Gonorrhea was diagnosed using Gram staining rather than nucleic acid amplification testing (NAAT), which is considered the diagnostic gold standard. Although Gram staining has high specificity (> 95%) and moderate-to-high sensitivity among symptomatic women, its performance is lower among asymptomatic individuals. Because NAAT was not available due to financial and laboratory resource constraints, some gonorrhea infections may have gone undetected, potentially leading to an underestimation of the true prevalence.

## Supplementary Information

Supplementary Material 1.

## Data Availability

The datasets used and/or analyzed during the current study are available from the corresponding author upon reasonable request.

## Abbreviations

AIDs: AIDS
AOR: Adjusted odds ratio
CI: Confidence interval
COR: Crude odds ratio
CSA: Central statistical agency (Ethiopia)
FSW: Female sex worker
HIV: Human immunodeficiency virus
KPC: Key population clinic
OR: Odds ratio
RPR: Rapid plasma regeneration
SBSW: Street-based sex worker
SPSS: Statistical package for the social sciences
STI: Sexually transmitted infection
WHO: World Health Organization
