# Supplementary material for: Laboratory-confirmed sexually transmitted infections and associated factors among street-based female sex workers in Southwest Ethiopia: a facility-based cross-sectional study
Source: BMC Public Health. 2026 Jul 14;26:2915. doi: 10.1186/s12889-026-28539-y (PMC13651165; doi:10.1186/s12889-026-28539-y)
Supplement: Supplementary file 1 [file 12889_2026_28539_MOESM1_ESM.pdf]

Patient Information and Informed Consent Form

You are invited to participate in a research study titled ***"Laboratory-Confirmed Sexually Transmitted Infections and Their Determinants among Women Entering Street-Based Sex Work before Age 18 in Southwest Ethiopia."*** The purpose of this study is to assess the prevalence of laboratory-confirmed sexually transmitted infections (STIs) and identify associated factors among women who began street-based sex work before the age of 18 years. If you agree to participate, you will be asked to complete an interviewer-administered questionnaire that takes about 20–25 minutes. The questions will include information about your socio-demographic characteristics, sexual and reproductive health history, condom use, substance use, experience of violence, and healthcare access. In addition, trained health professionals for STI testing will collect a biological sample (such as blood and/or vaginal swab). Some questions may be sensitive, and you may skip any question you do not wish to answer. Minor discomfort may occur during sample collection. There is no financial payment for participation; however, you will receive free STI testing and appropriate referral for treatment if needed. All information collected will be kept strictly confidential. Your name will not be recorded; instead, a code number will be used. Data will be stored securely and used only for research purposes. Your participation is voluntary, and you may refuse or withdraw at any time without any penalty or loss of services. By signing or providing your thumbprint below, you confirm that you have understood the information provided and voluntarily agree to participate in this study.

Do you agree with this? ___________(A). YES (B). NO

Participant ID: ____________________Signature/Thumbprint: __________ Date: __________

Data collector Name: ______________Signature: ____________________ Date: __________

ANNEX 1.

Table 8: Socio-Demographic Characteristics Participants

S.no	Categories/Variables	Answers	Skip
101	Age (completed years)	_________years	
102	Current residence:	A) Jimma B).Agaro	
103	Place of birth	A) Urban B) Rural	
104	Marital Status	A. Never married<br>B. Married<br>C. Divorced<br>D. Widowed	
105	Educational Status:	A. Cannot read and write<br>B. Can read and write<br>C. Primary<br>D. Secondary & Above	
106	Religion:	A) Orthodox<br>B) Muslim<br>C) Protestant<br>D) Catholic<br>E) Other	
107	Average monthly income (ETB):	_____ETB	
108	Age at first sex work engagement:	_______in Years	
109	Did you enter sex work before age 18?	A. Yes B. No	
110	Main reason for entering sex work:	A) Poverty<br>B) Family conflict<br>C) Orphan hood<br>D) Peer influence<br>E) Forced/trafficked<br>F) Other_______	
111	Who influenced your entry?	A) Self-decision<br>B) Friend<br>C) Brokers<br>D) Partner	
201	History of physical violence before age 18?	Yes B. No	
202	History of sexual abuse before age 18?	Yes B. No	

Table 9*: Sexual Behavior of Participants.*

S.no	Categories/Variables	Answers	Skip
303	Age at first sexual intercourse:	______	
304	Number of clients in last 7 days:	______	
305	Average number of clients per day:	______	
306	Condom use with Clients	A) Always<br>B) Sometimes<br>C) Never	
307	Condom breakage in last 6 months?	A. Yes B. No	

Table 10*: Substance Use of Participants*

S.no	Categories/Variables	Answers	Skip
401	Alcohol use:	A) Daily<br>B) Weekly<br>C) Occasionally<br>D) Neve	
402	Alcohol use before sex:	A. Yes B. No	
403	Khat chewing:	A) Daily<br>B) Weekly<br>C) Occasionally<br>D) Neve	
404	Drug use (hashish, shisha, others):	A. Yes B. No	

Table 11*: Reproductive Health History Of Participants*

S.no	Categories/Variables	Answers	Skip
501	Ever tested for HIV?	Yes B. No	
502	STI symptoms in last 12 months:	A) Vaginal discharge<br>B) Genital ulcer<br>C) Lower abdominal pain<br>D) Burning urination<br>E) No symptoms	
503	Visited health facility in last 12 months?	A. Yes B. Yes	
504	Received STI screening in last year?	A. Yes B. Yes	
505	Barriers to accessing care:	A) Stigma<br>B) Cost<br>C) Distance<br>D) Fear<br>E) None	

Table 12: Structural & Violence Factors

S.no	Categories/Variables	Answers	Skip
601	Ever-Experienced Sexual violence?	A. Yes B. NO	
602	Ever Experienced Psychical Violence?	A. Yes B. NO	

Table 13: Laboratory Results (To Be Completed By Clinician)

S.NO	Test	Result	
701	HIV	☐ Positive ☐ Negative	
702	Syphilis (RPR/TPHA)	☐ Positive ☐ Negative	
703	Gonorrhea	☐ Positive ☐ Negative	
704	Chlamydia	☐ Positive ☐ Negative	
705	Trichomonas	☐ Positive ☐ Negative	

THANK YOU!
